# Incidence and risk factors analysis for mortality after total knee arthroplasty based on a large national database in Korea

**DOI:** 10.1038/s41598-021-95346-3

**Published:** 2021-08-04

**Authors:** Ho-Jun Choi, Han-Kook Yoon, Hyun-Cheol Oh, Ju-Hyung Yoo, Chong-Hyuk Choi, Jin-Ho Lee, Sang-Hoon Park

**Affiliations:** 1 Department of Orthopaedic Surgery, National Health Insurance Service Ilsan Hospital, 100 Ilsan-ro, Ilsandong-gu, Goyang, 10444 Republic of Korea; 2 Department of Orthopedic Surgery, Gwangmyeong-Sungae Hospital, Gwangmyeong-si, Republic of Korea; 3 Department of Orthopedic Surgery, Severance Hospital, Yonsei University College of Medicine, Seoul, Republic of Korea

**Keywords:** Diseases, Health care, Medical research, Risk factors

## Abstract

This study aimed to analyze the rates and risk factors of postoperative mortality among 560,954 patients who underwent total knee arthroplasty (TKA) in Korea. The National Health Insurance Service-Health Screening database was used to analyze 560,954 patients who underwent TKA between 2005 and 2018. In-hospital, ninety-day, and one-year postoperative mortality, and their association with patient’s demographic factors and various comorbidities (ie., cerebrovascular disease, congestive heart failure, and myocardial infarction) were assessed. In-hospital, ninety-day and one-year mortality rates after TKA were similar from 2005 to 2018. The risk of in-hospital mortality increased with comorbidities like cerebrovascular disease (hazard ratio [HR] = 1.401; 95% confidence interval [CI] = 1.064–1.844), congestive heart failure (HR = 2.004; 95% CI = 1.394 to 2.881), myocardial infarction (HR = 2.111; 95% CI = 1.115 to 3.998), and renal disease (HR = 2.641; 95% CI = 1.348–5.173). These co-morbidities were also independent predictors of ninety-day and one-year mortality. Male sex and old age were independent predictors for ninety-day and one-year mortality. And malignancy was risk factor for one-year mortality. The common preoperative risk factors for mortality in all periods were male sex, old age, cerebrovascular disease, congestive heart failure, myocardial infarction, and renal disease. Malignancy was identified as risk factor for one-year mortality. Patients with these comorbidities should be provided better perioperative care.

## Introduction

Total Knee Arthroplasty (TKA) is performed in the advanced stage of osteoarthritis. It is a cost-effective treatment that has successful outcomes, which reduce pain and improve the quality of life for patients with advanced knee osteoarthritis^1–4^. With an increase in the number of patients with degenerative arthritis due an increase in the aging population, the number of patients undergoing total knee arthroplasty (TKA) for degenerative knee arthritis is gradually increasing worldwide. Obesity is known to be a major risk factor for knee arthritis^5,6^; this is also a reason for the increase in number of patients undergoing TKA. Between 2000 and 2014, the estimated annual numbers of primary TKA increased by 148% in the United States^7^. More than 700,000 cases of TKA are performed per year in the United States, and it is estimated that the number will increase to 3.5 million cases by 2030^2,3,8–11^. In Korea, the rates of primary TKA increased by 407% and the rates of revision TKA increased by 267%, between 2001 and 2010 respectively^12^.

TKA relieves pain with improvement in function; however, it is associated with an increase in fatal complications such as mortality^13,14^. TKA is usually performed in elderly patients above 65 years of age. The rate of TKA in those aged 50 years was 0.68% among the total population of the United States in 2010; however, it increased to 2.92% at 60 years, 7.29% at 70 years, and 10.38% at 80 years of age^8^. In Korea between 2001 and 2010, the highest rate of people undergoing TKA was observed in the age group of 65 to 74 years, and the highest increase in rate was seen in the age group of 75 to 84 years^12^. In elderly patients, comorbidities can aggravate after surgery depending on the general condition, and in severe cases, it might lead to death. In the case of patients with comorbidities, the risk of mortality was reported to be high^15^. However, there are few publications about the comparative analyses of risk, or large-scale studies. Moreover, the risk of mortality associated with each comorbidity is also unclear. Therefore, we aimed to analyze the incidence and risk factors for mortality after TKA in Korea through a large nationwide data research.

## Results

Total 560,954 cases of primary and revision TKAs performed between 2005 and 2018 in Korea were enrolled in this study. From 13,880 cases in 2005 to 60,558 cases in 2018, the number of patients undergoing TKA has increased over the years. In this study, the in-hospital mortality rate after TKA from 2005 to 2018 remained unchanged at 0.04%, and the ninety-day were similar from 0.17% to 0.15% and one-year mortality rates were also similar from 0.78 to 0.54% (Table 1).

**Table 1:** Mortality rates for total knee arthroplasty.

Year	Patients (n)	In-hospital mortality		90-day mortality		1-year mortality	
		n	%	n	%	n	%
2005	13,880	5	0.04	24	0.17	108	0.78
2006	20,067	13	0.06	39	0.19	155	0.77
2007	25,916	16	0.06	43	0.17	201	0.78
2008	29,980	21	0.07	63	0.21	225	0.75
2009	34,933	22	0.06	89	0.25	299	0.86
2010	39,007	18	0.05	85	0.22	297	0.76
2011	40,291	17	0.04	106	0.26	338	0.84
2012	43,171	28	0.06	108	0.25	349	0.81
2013	43,298	19	0.04	97	0.22	323	0.75
2014	44,045	21	0.05	83	0.19	317	0.72
2015	49,408	16	0.03	75	0.15	334	0.68
2016	57,580	28	0.05	115	0.20	390	0.68
2017	58,820	17	0.03	99	0.17	400	0.68
2018	60,558	25	0.04	92	0.15	325	0.54
Overall	560,954	266		1118		4061

1. Factors affecting one-year postoperative mortality after TKA

After TKA, the number of one-year postoperative deaths were 4061. Table 2 shows the results of a series of multivariable analyses using the Cox model. Among the patient's demographics, Male sex and old age was statistically significant effect on one-year mortality. The impact of AIDS, chronic pulmonary disease, diabetes, hemiplegia, mild liver disease, peripheral vascular disease, peptic ulcer disease, and rheumatologic disease on the risk of mortality was not statistically significant Some comorbidities were associated with an increased risk of mortality (Table 2). Cerebrovascular disease (HR = 1.27; 95% CI = 1.18 to 1.37, *P* < 0.0001), congestive heart failure (HR = 1.52; 95% CI = 1.38 to 1.68, *P* < 0.0001), dementia (HR = 1.28; 95% CI = 1.13 to 1.43, *P* < 0.0001), moderate or severe liver disease (HR = 4.34; 95% CI = 2.82 to 6.68, *P* < 0.0001), any malignancy including leukemia and lymphoma (HR = 2.97; 95% CI = 1.11 to 7.93, *P* = 0.0295), metastatic solid tumor (HR = 2.57; 95% CI = 1.07 to 6.19, *P* = 0.0352), myocardial infarction (HR = 1.53; 95% CI = 1.28 to 1.83, *P* < 0.0001), and renal disease (HR = 1.92; 95% CI = 1.58 to 2.34, *P* < 0.0001) were associated with death within one year of TKA.

**Table 2:** Cox proportional hazard models of one-year postoperative mortality by variables.

Variables	N	Multivariable analysis	*P*-value
		Hazard ratio (95% CI)	
**Sex**			
Male	1047	2.45 (2.28–2.62)	< 0.0001
Female	3014	1 (reference)	
**Age**			
50–59 yr	98	1 (reference)	
60–69 yr	767	1.55 (1.26–1.91)	< 0.0001
70–79 yr	2262	3.32 (2.71–.07)	< 0.0001
≥ 80 yr	934	7.30 (5.91–9.00)	< 0.0001
**AIDS/HIV**			
No	4060	1 (reference)	
Yes	1	0.57 (0.08–4.06)	0.5755
**Cerebrovascular disease**			
No	2964	1 (reference)	
Yes	1097	1.27 (1.18–1.37)	< 0.0001
**Congestive heart failure**			
No	3605	1 (reference)	
Yes	456	1.52 (1.38–1.68)	< 0.0001
**Chronic pulmonary disease**			
No	3033	1 (reference)	
Yes	1028	1.00 (0.93–1.08)	0.9297
**Dementia**			
No	3744	1 (reference)	
Yes	317	1.28 (1.13–1.43)	< 0.0001
**Diabetes without chronic complication**			
No	3120	1 (reference)	
Yes	947	0.86 (0.79–0.93)	0.0002
**Diabetes with chronic complication**			
No	3384	1 (reference)	
Yes	677	0.91 (0.83–1.00)	0.0513
**Hemiplegia or paraplegia**			
No	3978	1 (reference)	
Yes	83	0.99 (0.80–1.24)	0.964
**Mild liver disease**			
No	2627	1 (reference)	
Yes	1434	1.03 (0.96–1.10)	0.4426
**Moderate or severe liver disease**			
No	4040	1 (reference)	
Yes	21	4.34 (2.82–6.68)	< 0.0001
**Any malignancy, Including leukemia and lymphoma**			
No	4057	1 (reference)	
Yes	4	2.97 (1.11–7.93)	0.0295
**Metastatic solid tumor**			
No	4056	1 (reference)	
Yes	5	2.57 (1.07–6.19)	0.0352
**Myocardial infarction**			
No	3936	1 (reference)	
Yes	125	1.53 (1.28–1.83)	< 0.0001
**Peripheral vascular disease**			
No	3490	1 (reference)	
Yes	571	1.04 (0.95–1.13)	0.4473
**Peptic ulcer disease**			
No	2177	1 (reference)	
Yes	1884	1.03 (0.97–1.09)	0.4674
**Rheumatologic disease**			
No	3138	1 (reference)	
Yes	923	1.03 (0.96–1.11)	0.4311
**Renal disease**			
No	3957	1 (reference)	
Yes	104	1.92 (1.58–2.34)	< 0.0001

(2) Factors affecting ninety-day postoperative mortality after TKA

After TKA, the number of deaths in ninety days was 1118. Table 3 shows the results of a series of multivariable analyses using the Cox model. Ninety-day postoperative mortality increased with age (60 to 69 years, 70 to 79 years, over 80 years) and mortality was also higher in male than in female (Table 3). The HR increased in patients with comorbidities such as cerebrovascular disease (HR = 1.84; 95% CI = 1.53 to 2.20, *P* = 0.0003), congestive heart failure (HR = 1.84; 95% CI = 1.53 to 2.20, *P* < 0.0001), moderate or severe liver disease (HR = 5.55; 95% CI = 2.29 to 13.41, *P* = 0.0001), myocardial infarction (HR = 1.91; 95% CI = 1.39 to 2.63, *P* < 0.0001), renal disease (HR = 2.49; 95% CI = 1.77 to 3.50, *P* < 0.001).

**Table 3:** Cox proportional hazard models of ninety-day postoperative mortality by variables.

Variables	N	Multivariable analysis	*P*-value
		Hazard ratio (95% CI)	
**Sex**			
Male	264	2.02 (1.75–2.32)	< 0.0001
Female	854	1 (reference)	
**Age**			
50–59 yr	23	1 (reference)	
60–69 yr	208	1.79 (1.16–2.76)	< 0.0001
70–79 yr	587	3.81 (2.50–5.78)	< 0.0001
≥ 80 yr	300	10.40 (6.79–15.94)	< 0.0001
**AIDS/HIV**			
No	1117	1 (reference)	
Yes	1	2.16 (0.30–15.40)	0.4412
**Cerebrovascular disease**			
No	819	1 (reference)	
Yes	299	1.84 (1.53–2.20)	0.0003
**Congestive heart failure**			
No	978	1 (reference)	
Yes	140	1.84 (1.53–2.20)	< 0.0001
**Chronic pulmonary disease**			
No	847	1 (reference)	
Yes	271	0.94 (0.82–1.07)	0.3409
**Dementia**			
No	1032	1 (reference)	
Yes	86	1.17 (0.93–1.46)	0.183
**Diabetes without chronic complication**			
No	886	1 (reference)	
Yes	232	0.84 (0.72–0.98)	0.0279
**Diabetes with chronic complication**			
No	954	1 (reference)	
Yes	164	0.90 (0.75–1.08)	0.2639
**Hemiplegia or paraplegia**			
No	1109	1 (reference)	
Yes	9	0.52 (0.27–1.00)	0.0513
**Mild liver disease**			
No	744	1 (reference)	
Yes	374	0.95 (0.83–1.07)	0.3916
**Moderate or severe liver disease**			
No	1117	1 (reference)	
Yes	1	5.55 (2.29–13.41)	0.0001
**Any malignancy, Including leukemia and lymphoma**			
No	1118	1 (reference)	
Yes	0		
**Metastatic solid tumor**			
No	1117	1 (reference)	
Yes	1	1.63 (0.23–11.62)	0.6265
**Myocardial infarction**			
No	1078	1 (reference)	
Yes	40	1.91 (1.39–2.63)	< 0.0001
**Peripheral vascular disease**			
No	961	1 (reference)	
Yes	157	1.08 (0.91–1.28)	0.411
**Peptic ulcer disease**			
No	622	1 (reference)	
Yes	496	0.98 (0.87–1.10)	0.7277
**Rheumatologic disease**			
No	873	1 (reference)	
Yes	245	1.00 (0.87–1.15)	0.996
**Renal disease**			
No	1083	1 (reference)	
Yes	35	2.49 (1.77–3.50)	< 0.0001

(3) Factors affecting in-hospital mortality after TKA

After TKA, the total number of in-hospital deaths were 266. Table 4 shows the results of a series of multivariable analyses using the Cox model. Old age over 70 years was demographic factor that increase the risk of in-hospital mortality after TKA (Table 4). The risk of mortality during hospitalization increased in those with cerebrovascular disease (HR = 1.40; 95% CI = 1.06 to 1.84, *P* = 0.0163), congestive heart failure (HR = 2.00; 95% CI = 1.39 to 2.89, *P* = 0.0002), myocardial infarction (HR = 2.11; 95% CI = 1.12 to 4.00, *P* = 0.0217), and kidney disease (HR = 2.64; 95% CI = 1.35 to 5.17, *P* = 0.0046).

**Table 4:** Cox proportional hazard models of In-hospital mortality by variables.

Variables	N	Multivariable analysis	*P*-value
		Hazard ratio (95% CI)	
**Sex**			
Male	42	0.95 (0.68–1.32)	0.751
Female	224	1 (reference)	
**Age**			
50–59 yr	9	1 (reference)	
60–69 yr	53	1.17 (0.58–2.37)	0.6666
70–79 yr	136	2.33 (1.18–4.59)	0.0147
≥ 80 yr	68	6.66 (3.30–13.44)	< 0.0001
**AIDS/HIV**			
No	268	1 (reference)	
Yes	0		
**Cerebrovascular disease**			
No	192	1 (reference)	
Yes	74	1.40 (1.06–1.84)	0.0163
**Congestive heart failure**			
No	231	1 (reference)	
Yes	35	2.00 (1.39–2.88)	0.0002
**Chronic pulmonary disease**			
No	209	1 (reference)	
Yes	57	0.83 (0.62–1.11)	0.2151
**Dementia**			
No	246	1 (reference)	
Yes	20	1.21 (0.77–1.90)	0.4156
**Diabetes without chronic complication**			
No	214	1 (reference)	
Yes	52	0.79 (0.57–1.09)	0.1514
**Diabetes with chronic complication**			
No	226	1 (reference)	
Yes	40	0.96 (0.67–1.38)	0.8193
**Hemiplegia or paraplegia**			
No	266	1 (reference)	
Yes	0		
**Mild liver disease**			
No	182	1 (reference)	
Yes	84	0.92 (0.71–1.20)	0.5373
**Moderate or severe liver disease**			
No	266	1 (reference)	
Yes	0		
**Any malignancy, Including leukemia and lymphoma**			
No	266	1 (reference)	
Yes	0		
**Metastatic solid tumor**			
No	265	1 (reference)	
Yes	1	6.75 (0.94–48.32)	0.0571
**Myocardial infarction**			
No	256	1 (reference)	
Yes	10	2.11 (1.12–4.00)	0.0217
**Peripheral vascular disease**			
No	229	1 (reference)	
Yes	37	1.14 (0.80–1.61)	0.4687
**Peptic ulcer disease**			
No	156	1 (reference)	
Yes	110	0.90 (0.70–1.14)	0.3648
**Rheumatologic disease**			
No	213	1 (reference)	
Yes	53	0.83 (0.61–1.12)	0.2271
**Renal disease**			
No	258	1 (reference)	
Yes	8	2.64 (1.35–5.17)	0.0046

## Discussion

TKA is the most commonly performed surgical procedure in patients with knee osteoarthritis, and the number of patients who underwent TKA has increased^8,16–18^. In the 13 years from 2005 to 2018, the rate of TKA in Korea increased by 436% from 13,880 cases to 60,558 cases. Mortality after TKA is rare, and this low rate makes it difficult to identify the risk factors associated with mortality. In our large-scale retrospective cohort study, preoperative risk factors for mortality were male sex, old age, comorbidities such as cerebrovascular disease, congestive heart failure, myocardial infarction, and renal disease.

In this study, In-hospital, ninety-day and one-year mortality rates after TKA were similar from 2005 to 2018. The results of mortality in our study were lower than those published in previous papers, e.g. Smith et al. reported a 30-day mortality of 0.08% between 2005 and 2011^19^. Memtsoudis et al. reported a cumulative in-hospital mortality rate of 0.35% between 1990 and 2004, among 6,901,324 patients^20^. This decrease in mortality might be due to the exclusion of high-risk patients from elective surgery; however, the reduction might also be due to advances in anesthesia techniques, surgical methods, and perioperative care^21,22^.

As reported in other studies, the mortality rate after TKA in elderly patients was increased in our study^23–25^. Old age patients reduce their resistance to the stress of surgery, and comorbidities such as cardiovascular disease, cerebrovascular disease and renal disease are more frequent. As in other studies, in this study, male reported a higher mortality rate after TKA than female^13,23^.

Increased in-hospital mortality and ninety-day postoperative mortality was found to be associated with cardiovascular causes such as cerebrovascular disease, myocardial infarction and renal disease. In addition, comorbidities such as chronic pulmonary disease, diabetes, hemiplegia, mild liver disease, peripheral vascular disease, peptic ulcer disease, and rheumatologic disease were found to have no effect on the risk of mortality. At one-year after TKA, any malignancy (HR = 2.97; 95% CI = 1.11 to 7.93, *P* = 0.0295) and metastatic solid tumor (HR = 2.57; 95% CI = 1.07 to 6.19, *P* = 0.0352) were additionally found to be a comorbidity associated with an increase in mortality, unlike that for in-hospital mortality and ninety-day postoperative mortality. This might be related to the progression of metastatic tumors over a period of time after surgery.

In this study, cardiovascular causes (congestive heart failure and myocardial infarction) were found to be related to mortality for all time periods including in-hospital mortality, ninety-day postoperative mortality, and one-year postoperative mortality. Cardiovascular disease (congestive heart failure and myocardial infarction) has been shown to be a common cause of mortality after TKA in several studies^19,23,26,27^. Singh, J.A. et al. reported that primary cardiac events were the major predictors for postoperative ninety-day mortality (Odds ratio [OR], 2.3; 95% CI, 1.3 to 4.1, *P* = 0.005)^26^. Hunt, L.P. et al. described myocardial infarction (Hazard ratio [HR], 3.46; 95% CI, 2.89 to 4.14, *P* < 0.0005) and congestive heart failure (Hazard ratio [HR], 3.41; 95% CI, 2.81 to 4.14, *P* < 0.0005) as a major risk factors for 45-day mortality^23^.

Renal disease was identified as a comorbidity associated with mortality in our study. Renal disease included chronic kidney disease, which is defined as a decrease in glomerular filtration rate and increase in albumin excretion. The effect of renal disease on mortality in patients who underwent TKA has been investigated in several studies^20,23,28–33^. In the meta-analysis of mortality, the hazard ratio of mortality after TKA was significantly higher in patients with chronic kidney disease (HR = 1.45; 95% CI = 1.02–2.05, *P* = 0.04)^31^. Hunt et al. reported that renal disease increased the risk of 45-day mortality after TKA in a study involving a large national registry (HR = 2.18; 95% CI = 1.76 to 2.69, *P* < 0.0005)^23^. The decrease in renal perfusion due to perioperative blood loss is expected to adversely affect the progression of renal disease and could increase the risk of mortality. Renal disease could also affect the risk of complications such as peri-prosthetic joint infection and rate of arthroplasty revision surgery^31,33^. Therefore, careful preoperative risk stratification is important in patients with renal disease.

Cerebrovascular disease appeared to be a significant risk factor for mortality at all time points after TKA in our study. Similar results have been reported in other studies. Memtsoudis, S.G. et al. reported that cerebrovascular disease increased the odds of in-hospital mortality by approximately four times^20^ Hunt et al. reported that cerebrovascular disease was associated with a three-fold increase in the relative risk of death within 45 days of TKA (HR = 3.35; 95% CI = 2.70–4.14, *P* < 0.0005)^23^. Bozic, K.J. et al. also reported that cerebrovascular disease increased the ninety-day postoperative mortality after TKA (HR = 1.49; 95% CI = 1.19. 1.87, *P* = 0.0005)^34^.

Several other studies have reported significant associations between mortality and other comorbidities. Some studies reported that diabetes without complications was a significant risk for mortality^20,23,25^ and liver disease increased the risk of mortality after TKA^23,34^. In this study, diabetes without chronic complication and moderate to severe liver disease increased the risk of ninety-day and one-year mortality. However, our study found no association between diabetes, liver disease, and in-hospital mortality after TKA.

Several limitations of this study should be noted. First, the limitation of this study is that the study period (2005–2018) was relatively short. As a result, it did not reflect the overall natural progression of OA and TKA, which require long-term follow-up. Before 2005, there was no data in the NHIS database, so there was a limit to the investigation for long term study period. Second, since the data are based on the health insurance service billing data code, some codes and data might have been missed during the billing process of each hospital. Since the study was based on data from the national registry, it was impossible to determine the severity of each disease according to the test value of each comorbidity. Moreover, it was not possible to analyze other factors that could affect complications such as body mass index (BMI), smoking, drinking, lifestyle, mental health, and the patients’ knee function before surgery, revision TKA. Future studies that investigate these factors such as socioeconomic factors in Korea that can affect the complications are required. Third, in this study, all-cause mortality was assessed and we were not able to investigated case-specific mortality. This study is significant as a large national database study, and although it can analyze some trends, it has the limitation of not being able to analyze cause-specific mortality.

## Conclusion

We analyzed the trend and specific baseline comorbidity associated with mortality after TKA. Preoperative risk factors for mortality were male sex, old age, cerebrovascular disease, congestive heart failure, any malignancy including leukemia and lymphoma, myocardial infarction, and renal disease. Patients with these comorbidities should be provided better perioperative care.

## Materials and methods

The Institutional Review Board of National Health Insurance Service Ilsan Hospital (NHIMC 2020-09-0001) approved this retrospective Health Insurance Portability and Accountability Act-compliant cohort study and waived the informed consent from the participants, because this study was expected to present no or minimal risk of harm to the participants, and all the data used were anonymized. All methods were performed in accordance with relevant guidelines and regulations. This study is retrospective cohort study using customized data provided by the National Health Insurance Service (National Health Insurance Service-HealthScreening; NHIS-HealS). In Korea there is an obligatory National Health Insurance system with universal coverage. NHIS-HealS database has reimbursement records from all medical institutions in Korea. Primary and revision TKAs were investigated by their principal procedure codes in the NHIS-HealS database. Among patients diagnosed with primary knee arthrosis (diagnostic codes : M170, M171), patients above 50 years of age who had been charged for hospitalization, and underwent primary TKA or revisional TKA between January 1, 2005 and December 31, 2018 were included in the study. Records with procedural codes of primary TKA (N0712, N2072, N2077) and revision TKA (N1712, N3712, N3717) were selected. Patients who underwent TKA due to post-traumatic osteoarthritis (diagnostic codes : M172,M173), secondary osteoarthritis (M174,M175), pyogenic arthritis (M00,M01), and reactive arthritis (M02) were excluded. Patients who had previously undergone total hip arthroplasty(THR) (procedural codes : N0711), and had undergone high tibial osteotomy (N0304) or unicompartmental knee arthroplasty (N2712) on the same knee were also excluded. A total of 560,954 patients were included.

In this case, we investigated all-cause mortality and cases of mortality were divided into groups according to the period from surgery to mortality (in-hospital mortality, ninety-day postoperative mortality, one-year postoperative mortality). Definition of ‘in-hospital mortality’ is the mortality cases within in-hospital period and definition of ‘ninety-day postoperative mortality’ is the mortality cases until postoperative 90 days, and definition of ‘one-year postoperative mortality’ means death between postoperative 0 day and 1 year. This was to confirm the difference between the period of death after TKA and to determine whether the effect of comorbidity differs by the period from surgery to mortality.

Patient demographic factors including age and sex, comorbidities were investigated. We used the International Classification of Diseases 10 codes reported in NHIS and defined the subgroup 17 as high-risk, in which a higher rate of mortality was expected, as originally proposed by Charlson et al.^35,36^. The comorbidities included for the analysis were AIDS, cerebrovascular disease, congestive heart failure, chronic pulmonary disease, dementia, diabetes without chronic complications, diabetes with chronic complications, hemiplegia or paraplegia, mild liver disease, moderate or severe liver disease, any malignancy including leukemia and lymphoma, metastatic solid tumor, myocardial infarction, peripheral vascular disease, peptic ulcer disease, rheumatoid disease, and renal disease.

For all analyses, SAS 9.4 (SAS Inc., Cary, NC, USA) was used. We performed multivariable Cox proportional hazards model analyses of the 17 comorbidities. Hazard ratios (HRs) and 95% confidence intervals (CIs) are presented. The level of significance was maintained at a *P* value < 0.05.

## Data Availability

All data during this study are not publicly available because all data have been deposited in the National Health Insurance Service-HealthScreening (NHIS-HealS) Database. But all data during this study are included in this article.
